# Associated Factors with Low Birth Weight in Dire Dawa City, Eastern Ethiopia: A Cross-Sectional Study

**DOI:** 10.1155/2019/2965094

**Published:** 2019-12-09

**Authors:** Alekaw Sema, Firehiwot Tesfaye, Yalelet Belay, Bezabh Amsalu, Desalegn Bekele, Assefa Desalew

**Affiliations:** ^1^Department of Midwifery, College of Medicine and Health Sciences, Dire Dawa University, Dire Dawa, Ethiopia; ^2^School of Medicine, College of Medicine and Health Sciences, Dire Dawa University, Dire Dawa, Ethiopia; ^3^Department of Public Health, College of Medicine and Health Sciences, Dire Dawa University, Dire Dawa, Ethiopia; ^4^School of Nursing and Midwifery College of Health and Medical Sciences, Haramaya University, Harar, Ethiopia

## Abstract

**Background:**

Low Birth Weight (LBW) is a serious public health concern in low- and middle-income countries. Globally, 20 million, an estimated 15% to 20% of babies were born with LBW, and, of these, 13% were in sub-Saharan Africa. Although the World Health Assembly targeted to reduce LBW by 30% by the end of 2025, little has been done on and known about LBW. To meet the goal successfully and efficiently, more research studies on the problem are vital. Hence, the aim of this study was to determine the prevalence and the associated factors of LBW in Dire Dawa city, eastern Ethiopia.

**Objective:**

The purpose of this study was to assess the prevalence and the associated factors of low birth weight in Dire Dawa City, eastern Ethiopia, 2017.

**Method:**

A cross-sectional study designed was conducted, and using a systematic sampling technique, 431 mothers who gave birth in the public hospitals in Dire Dawa city from July 01 to August 30, 2018, were selected. Stillbirth and infants with birth defects were excluded from the study. Well-trained data collectors collected the data using a structured questionnaire which was pretested. The data were analyzed using SPSS Version 22.0. The Adjusted Odds Ratio (AOR) with 95% confidence interval (CI) was applied in multivariate logistic regression models, and *p* value less than 0.05 was considered as statistical significant.

**Result:**

The prevalence of low birth weight was 21%. Not received nutritional counseling during antenatal care (AOR = 2.03, 95% CI: 1.01, 4.06), preterm birth (AOR = 18.48, 95% CI: 6.51, 52.42), maternal smoking (AOR = 3.97, 95% CI: 1.59, 9.88), and height of the mother less than 150 cm (AOR = 3.54, 95% CI: 1.07, 11.76) were significantly associated with Low birth weight.

**Conclusion:**

There was a high prevalence of low birth weight in the study area. Effective dietary counseling and additional diet, implementing proven strategies to prevent preterm birth and avoid smoking during pregnancy might decrease the low birth weight and then enhance child survival.

## 1. Introduction

The burden of low birth weight (LBW) is a serious public health concern in low- and middle-income countries [1, 2]. Globally, more than 20 million (an estimated 15 to 20%) newborns were LBW, and 13% of them were in sub-Saharan Africa (SSA) in 2015 [3–5]. These babies were more likely to die during their first month of life, and those who survived would face lifelong consequences including a higher risk of stunting, low intelligent quotient (IQ), and adult-onset chronic conditions such as obesity, hypertension, and diabetes mellitus [5–10]. Furthermore, LBW is a significant determinant of infant and childhood morbidity including neurodevelopment impairment such as mental retardation, cerebral palsy, and learning disability [1, 5, 11, 12]. The under-five mortality rate decreased from 91 deaths per 1,000 live births in 1990 to 43 per 1,000 in 2015. However, the decline in neonatal mortality from 1990 to 2015 was slower than that of postneonatal under-five mortality [13].

The World Health Organization (WHO) defines LBW as a birth weight of less than 2,500 grams [2, 4]. LBW continues to be an unfinished agenda because it is one of the poor pregnancy outcomes; it is a good indicator of the health of an infant and is a principal factor that determines the infant's physical, survival, and mental growth. It also indicates the past and present health status of the mothers that have caught the attention of WHO [2, 4, 14–16] and still remains the single most important cause of child morbidity and mortality, especially in SSA, where most LBW babies are born [2, 17].Hence, by the end of 2025, World Health Assembly set a policy target to reduce LBW by 30%. Different strategies have been implemented to reduce newborns with LBW with the packages of care provided at the prenatal, antenatal, intranatal, and postnatal period [1, 3, 5, 11]. This would translate into a 3.9% relative reduction per year between 2012 and 2025 and a reduction from approximately 20 million to about 14 million newborns with LBW at birth [3, 18].

In multiple studies, different factors have been identified as determinant for LBW, and among these are young maternal age at pregnancy, birth order, the family's income, maternal undernutrition, maternal underweight, pregnancy-related complications, preterm birth, chronic medical illness, multiple pregnancies, history of previous LBW, insufficient prenatal care, and maternal smoking [19–22].

In Ethiopia, the issue of LBW and the factors influencing it have not received much attention. However, the country is with very high neonatal mortality due to factors associated with LBW and are one of the critical issues that cause babies to suffer from short-term and long-term health consequences and mortality [23–26].

Although LBW has been reported to account for perinatal morbidity and mortality and extensively explored in developed countries, in developing countries, including Ethiopia, few studies are available on LBW. In order to prevent LBW, an understanding of its main modifiable risk factors is essential, so that health managers and practitioners may use for plan strategies and implement appropriate interventions toward promoting health [22, 27]. Hence, the aim of this study was to determine the prevalence and the associated factors of LBW in Dire Dawa City Administration, eastern Ethiopia.

## 2. Methods and Materials

### 2.1. Study Setting, Design, Population, and Period

An institutional-based cross-sectional study design was conducted in Dire Dawa City Administration. It is located 515 kilometers away from Addis Ababa, the capital of Ethiopia. According to the 2007 Ethiopian census, an estimated 3,96,423 people were living in the administration. It has achieved 100% primary health care access. In terms of the distribution of health facilities, there are 2 governmental and 4 private hospitals, 8 health centers, 5 higher clinics, and 12 medium clinics in the city. Mothers who gave birth in Dilchora Referral Hospital and Sabina Primary Hospital from July to August 2018 were included. According to the Dire Dawa City Administration's health office report, approximately about 2000 live births happened every two months in the administration and 58.7% of delivery took place in the health facilities (26). The two hospitals were included because more than two-thirds (1260) of the delivery takes place in these facilities. Stillbirth and infants with birth defects were not included in this study.

### 2.2. Sample Size and Sampling Procedures

The sample size was determined using a single population proportion formula (*n* = (*Zα*/2)^2^*pq*/*d*^2^) by considering the proportion of LBW in eastern Ethiopia 21.9% [28] and using 95% CI, 4% marginal error, and 5% of nonresponse rate. The final sample size was 431. Moreover, the double population proportion formula was used to determine the sample size for the factors associated with LBW. Also, this was calculated for some of the associated factors obtained from different literatures using Epi Info statistical software version 7 with the following assumptions: confidence level = 95%, power = 80%, the ratio of unexposed to exposed almost equivalent to 1 not received dietary counseling 34% (19). This yields 144 participants. Finally, we selected the largest sample size from the first objective, which was 431 samples. According to the hospital's delivery report, about 1,260 mothers give birth per two months. Hence, the study subjects were selected using a systematic sampling technique. The sampling interval (*K*) was three. The initial mother was employed using the lottery method. When the selected study subject did not fulfill the inclusion criteria, the subsequent mother was included.

### 2.3. Data Collection Tool and Quality Control

The data were collected through a face-to-face interview and using a questionnaire which was an adapted and modified form different works of the literature and prepared originally in English, translated into local languages (Amharic, Afan Oromo, and Somali) and then translated back into English for checking the consistency by different language expertise. Trained midwives and nurses working in the labour ward conducted the interview and anthropometric measurements. The weight of the newborns was measured within the first hour of birth using a balanced Seca scale. The measurement scale was always checked and calibrated before weighing each newborn. Maternal height was measured against a wall using a height scale to the nearest centimeter, and maternal weight was measured by using a beam balance to the nearest kilogram. To ensure the quality of the data, a two-day intensive training was given for all the supervisors and the data collectors. The data collection process was undertaken with frequent monitoring and supervision. Finally, double data entry was done to check the consistency of the data and minimize the entry errors.

### 2.4. Operational Definition

Birth weight: the first weight of the newborns measured within the first hour after birth. Low birth weight was for those newborns who weighed less than 2500 g, while those newborns with a birth weight of 2500 g and above were considered of normal birth weight.

### 2.5. Data Processing and Analysis

The data were coded, entered into EPI data Version 3.1, and exported to SPSS Version 22.0 software for analysis. Then, they were summarized and presented using descriptive statistics. The outcome variables were coded as “1” for LBW whereas “0” for others. The association between the outcome variables (i.e., LBW) and the independent variables was analyzed using a binary logistic regression model. The covariates which had a *p* value <0.2 were retained and entered into the multivariable logistic regression analysis. Hosmer and Lemeshow goodness-of-fit tests were used to assess whether the necessary assumptions were fulfilled. Adjusted odds ratio (AOR) with 95% confidence intervals (CI) was used. A *p* value <0.05 was considered statistically significant.

### 2.6. Ethical Considerations

Before the data collection, ethical permission was obtained from the ethical review committee of the College of Medicine and Health Science in Dire Dawa University, and informed written consent was obtained from the participants before conducting the interview and the measurement.

## 3. Results

### 3.1. Maternal Sociodemographic Characteristics

Four hundred and twenty mothers were included in the study, with a response rate of 97.40%. The mean (±SD) age of the mothers was 27.4 (±4.98) years. More than half of the study participants, 245 (58.3%), were in the age group of 20–34 years, followed by the age group of above 30 years, 142 (33.8%). Three hundred and seventy-six (89.5%) mothers were married. Regarding religious distribution, 242 (57.6%) were Muslim, and 119 (28.3%) were Orthodox Christians (Table 1).

### 3.2. Past Obstetric Characteristics of Mothers

Out of the total participants, 245 (53.3%) had 2–4 children. About a quarter of the mothers (26.1%), 67(23.3%), and 41(14.3%) had a history of abortion, stillbirth, and previous LBW, respectively.

### 3.3. Current Obstetric History of Mothers

Among the respondents who delivered in governmental hospitals, 320 (76.2%) had planned pregnancy. Most of the respondents, 395 (95%), had singleton babies. Two hundred and ninety-six (70.5%) of the mothers had attended antenatal care (ANC) visits. One hundred and thirty-four (45.3%) of them had less than four ANC visits. More than half (54.7%) of the ANC attendants had four or more ANC follow-ups. Among the ANC attendants, 277 (93.6%) had utilized iron, and of them, 32.1% had taken iron for four or more months during pregnancy. About 241 (81.4%) of the mothers had been vaccinated for tetanus toxoid, of whom 48% were vaccinated only once. Three hundred and ninety-one (93.1%) of the respondents were human immunodeficiency virus (HIV) negative, while only 29 (6.9%) were HIV positive. Among the respondents, 191 (45.5%) had used contraceptive methods preceding the current pregnancy. The level of hemoglobin among the mother was below 7, 8–11, and 12 or above gm/dl for 3.8%, 57.45%, and 38.8%, respectively (Table 2).

### 3.4. Prevalence of Low Birth Weight

**Figure 1:**
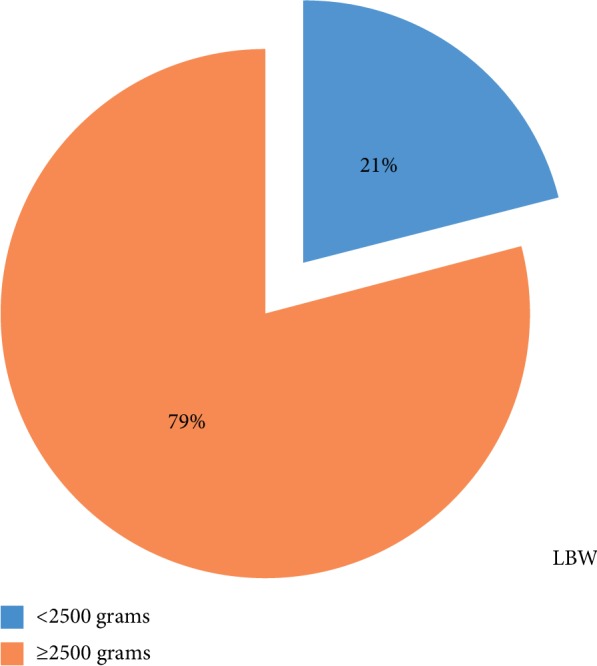
Prevalence of LBW among newborns delivered in Dire Dawa government hospital, eastern Ethiopia, 2018 (*n* = 420).

From the total newborns, 21% had LBW while 79% were at or above 2500 grams. The mean birth weight of the newborns was 3002 gm ± 709.2 grams (Figure 1).

### 3.5. Characteristics of the Newborn and Labour

Three hundred and eighty-three (91.2%) of the mothers had delivered at or after 37 weeks of gestation. Three hundred and sixty-nine (87.9%) of the newborns had an APGAR (appearance, pulse, grimace, respiration) score of seven and above, whereas 51 (12.1%) had less than seven within the first minute. More than half (53.3%) of the newborns were female. Two hundred and fifteen (51.2%) of the mothers had given birth through the spontaneous vaginal delivery (Table 3). The types of complications among the respondents were pregnancy-induced hypertension 100 (23.8%), antepartum hemorrhage 120 (28.7%), premature rupture of membrane 69 (16.5%), RH-iso-immunization 36 (8.5%), and urinary tract infection 77 (18.3%).

### 3.6. Anthropometric and Behavioral Characteristics

Most of the respondents, 216 (92.7%), had a weight of 50 kilograms and above for this pregnancy. Three hundred and ninety-nine (95%) of them had a height of 150 centimeter and above. More than two-thirds (72.1%) of the mothers' Mid-Upper Arm Circumference (MUAC) was above 22 centimeters. Most of the mothers did not smoke (92.9%) and drink alcohol (95%) during pregnancy (Table 4).

### 3.7. Factors Associated with LBW

In the multivariable analysis, the factors significantly associated with LBW were not received dietary counseling during ANC, preterm birth, maternal smoking during pregnancy, and maternal height. The mothers who did not receive nutritional counseling during ANC follow-up were 2 times (AOR = 2.03, 95% CI: 1.01, 4.06) more likely to give LBW baby compared with their counterparts. Furthermore, the neonates who were delivered before 37 weeks of the gestational age were 18 times (AOR = 18.48, 95% CI: 6.51, 52.42) more likely to be LBW compared with the neonates delivered at or above 37 weeks of gestation. The pregnant women who were smoking during pregnancy were nearly 4 times (AOR = 3.97, 95% CI: 1.59, 9.88) more likely to deliver LBW babies compared with their counterparts. In addition, the mothers whose height was less than 150 centimeters (AOR = 3.54, 95% CI: 1.07, 11.76) had about four times the chance of delivering LBW babies than those with height greater than 150 centimeters (Table 5).

## 4. Discussion

In the present study, the prevalence of LBW was 21% (95% CI: 17.1%, 24.8%). In the multivariable analysis, not received dietary counseling during ANC, preterm birth, maternal smoking during pregnancy, and maternal height were significantly associated with LBW. The mean weight of the newborns was 3002 ± 709.2 grams. The prevalence of LBW in this study was higher than the ones found in studies conducted in Italy (11.8%), Brazil (7.6%), Iran (5.2%), Kenya (12%), and Malaysia (13.96%) [29–33]. This difference may be due to the socioeconomic disparity and the level of care given to prevent preterm labour in developed countries. In addition, this difference might be because pregnant women in those countries have better disease screening and prevention and they might get better nutrients before and after pregnancy. This finding also higher than studies conducted in another setup in Ethiopia ranged from 9.9% to 17.4% [34–37]. This difference may be due to the inclusion of both public and government health facilities in previous studies. Moreover, mothers who were economically well may use private facilities and had low magnitude of LBW.

However, the finding of this study is consistent with the ones reported from Uganda (25.5%), Tanzania (21%), and Ethiopia [15, 38]. It is also comparable with those found in Bahirdar, Jimma, and eastern Ethiopia, where the prevalence of LBW was 21.2%, 22.5%, and 21.9%, respectively [19, 28, 39]. This might be due to the similarity in the study setting, methodology, and the availability of service delivery and socioeconomic condition between those countries and Ethiopia.

The mothers who did not receive nutritional counseling during the time of ANC follow-up were more likely to deliver LBW babies compared with the mothers who had received nutritional counseling during ANC follow-up. This is the fact that counseling about nutrition has increased the awareness of pregnant women on nutrition at the time of pregnancy [40]. This finding is consistent with the study results in Japan and Ethiopia [20, 39–41]. In fact, receiving dietary counseling and taking an adequate and balanced diet had a positive impact on the mothers as well the fetus's weight during pregnancy. Moreover, healthy and optimal intrauterine fetal growth relies heavily on maternal nutrient status [42].

As per this study, preterm newborns were more likely to be LBW compared with newborns delivered at or above 37 weeks of gestation. The finding of this study was congruent with those findings of studies conducted in Uganda, Kenya, Zimbabwe, and Ethiopia [19, 21, 29, 33, 34, 37–39, 41, 43, 44]. This may be due to the maximum weight gain for the newborns achieved in the third trimester of pregnancy and preterm neonates who are immature and have low physical developmental levels. In addition, this might be due to the fact that an immature newborn is more likely to be LBW and have respiratory problems and different complications [15, 45–47].

Smoking habits during pregnancy were significantly associated with LBW. The newborns who were delivered by mothers who had always smoked during pregnancy were more likely to be LBW compared with those from nonsmoking mothers. This is in line with the findings in Switzerland and Tanzania [17, 22, 31, 48]. This might be due to the fact that maternal smoking increases the risk of preterm birth and affects intrauterine fetal growth [22, 29, 49].In addition, we found an association between maternal height and LBW. The mothers who were less than 150 centimeters in height were about 3.5 times more likely to give LBW babies as compared with their counterparts. This finding is in agreement with different study results in Japan, China, and Ethiopia [22, 48, 49].

### 4.1. Strength and Limitations

This study used a probability sampling procedure to select the study participants. We used the questionnaire method and anthropometric measurement (height and weight) to assess LBW. Since the study was cross sectional, it was not possible to strongly demonstrate cause and effect relationships. We used self-reporting (interview response) which might have social desirability bias. Some questions also required the participant's recall, which could have affected the results, as most of them could have forgotten.

## 5. Conclusion

The prevalence of LBW in the study area was high. The mothers who did not receive nutritional counseling during ANC, preterm birth, babies from smoking mothers, and maternal height were associated with LBW. Effective dietary counseling and additional diet, implementing proven strategies to prevent preterm birth and avoid smoking during pregnancy, would improve the birth weight of the newborn, enhance child survival, and reduce child morbidity and mortality.

## Figures and Tables

**Table 1:** Sociodemographic characteristics of mothers delivered at governmental hospitals of Dire Dawa City, eastern Ethiopia, 2018 (*n* = 420).

Variables	Frequency	Percent (%)
*Maternal age*		
15–19	18	4.3
20–24	108	25.7
25–29	152	36.2
30 and above	142	33.8

*Marital status*		
Married	376	89.5
Single	25	7.7
Others	19	4.4

*Religion*		
Muslim	242	57.6
Orthodox	119	28.3
Protestant	54	12.9
Others	5	1.2

*Maternal educational status*		
Unable to read and write	80	19
Able to read and write	85	20.2
Attend primary school	121	28.8
Attended secondary school	48	11.4
College and above	86	20.5

*Maternal occupational status*		
Housewife	169	40.2
Merchant	130	31
Government employee	72	17.1
Nongovernmental employee	49	11.6

*Residence*		
Urban	306	72.9
Rural	114	27.1

*Family size*		
<4	293	69.8
4-5	56	13.3
>5	71	16.9

**Table 2:** History of current pregnancy among mothers who gave birth in public hospitals of Dire Dawa City, eastern Ethiopia, 2018 (*n* = 420).

Variables	Frequency	Percent (%)
*Type of pregnancy*		
Single	395	94.0
Multiple	25	6.0

*Time of starting ANC visit (296)*		
Before 4 months	137	46.3
At or after 4 months	159	53.7

*Number of ANC visit (296)*		
Less than 4 times	134	45.3
Four and above	162	54.7

*Maternal HIV status*		
Positive	29	6.9
Negative	291	93.1

*Counseling on nutrition at ANC*		
Yes	268	68.6
No	152	31.4

*Have you taken an additional diet?*		
Yes	288	68.6
No	132	31.4

*Hemoglobin level*		
Less or equal to 7 gm/dl	16	3.8
8–11 gm/dl	242	57.45
Greater than or equal to 12 gm/dl	163	38.8

**Table 3:** Characteristics related to newborn and delivery among mothers who gave birth in Dire Dawa government hospital, eastern Ethiopia, 2018 (*n* = 420).

Variables	Frequency	Percentage (%)
*Gestational*		
<37 weeks	37	8.8
≥37 weeks	383	91.2

*APGAR score within the 1* ^*st*^ * minute*		
<7/10	51	12.1
≥7/10	369	87.9

*Sex of the neonate*		
Male	195	46.7
Female	224	53.3

*Fetal presentation*		
Cephalic	322	76.7
Breach	98	23.3

*Mode of delivery*		
Spontaneous vaginal delivery	215	51.2
Instrumental	135	32.1
Caesarian section	70	16.7

*Complication during pregnancy*		
Yes	164	39
No	256	61

**Table 4:** Anthropometric and behavioral characteristics of mothers who gave birth in Dire government hospital, eastern Ethiopia, 2018 (*n* = 420).

Variables	Frequency	Percent
*Weight of the mother before pregnancy*		
<50 kilo gram	17	7.3
≥50 kilo gram	216	92.7

*Height of the mother*		
<150 centimeter	21	5
≥150 centimeter	399	95

*MUAC*		
≤22 centimeter	117	27.9
>22 centimeter	303	72.1

*Ever smoked during the current pregnancy*		
Yes	30	7.1
No	390	92.9

*Any person smoked in the surroundings*		
Yes	78	18.6
No	342	81.4

**Table 5:** Factors associated with low birth weight among neonates delivered in Dire Dawa in Dire Dawa government hospital, eastern Ethiopia, 2018 (*n* = 420).

Variables	LBW		COR (95% CI)	AOR (95% CI)	*p* value
	Yes	No			
*Residence*					
Urban	49 (16.0)	257 (84.0)	1	1	
Rural	39 (34.2)	75 (65.8)	2.7 (1.67, 4.47)	0.85 (0.39, 1.86)	0.77

*Level of education*					
No formal education	50 (30.3)	115 (69.7)	2.5 (1.54, 4.0)	1.55 (0.77, 3.13)	0.22
Formal education	38 (14.9)	217 (85.1)	1	1	

*Pregnancy intention*					
Planned	52 (16.2)	268 (83.8)	1	1	
Unplanned	36 (36.0)	64 (64.0)	2.9 (1.75, 4.80)	1.39 (0.68, 2.84)	0.35

*Nutritional counseling*					
Yes	40 (14.9)	228 (85.1)	1	1	
No	48 (31.6)	104 (68.4)	1.6 (2.63, 4.25)	**2.03 (1.01, 4.06)**	**0.047**

*Contraceptive use*					
Yes	26 (13.6)	165 (86.4)	1	1	
No	62 (27.1)	167 (72.9)	2.4 (1.42, 3.90)	1.34 (0.67, 2.67)	0.40

*Pregnancy complication*					
Yes	47 (28.7)	117 (71.3)	2.1 (1.30, 3.39)	0.72 (0.36, 1.45)	0.36
No	41 (16.0)	215 (84.0)	1	1	

*ANC follow-up*					
Yes	46 (15.5)	250 (84.5)	1	1	
No	42 (33.9)	82 (66.1)	2.8 (1.71, 4.53)	0.97 (0.39, 2.38)	0.94

*Hemoglobin level*					
Anemic	64 (24.9)	193 (75.1)	1.9 (1.15, 3.22)	1.25 (0.67, 2.36)	0.48
Normal	24 (14.7)	139 (85.3)	1	1	

*Mode of delivery*					
Vaginal	52 (18.2)	233 (81.8)	1	1	
Caesarian section	36 (26.7)	99 (73.3)	1.6 (1.0, 2.65)	1.06 (0.54, 2.09)	0.87

*MUAC*					
Less than 23 cm	40 (34.2)	77 (65.8)	2.7 (1.69, 4.50)	1.61 (0.86, 3.03)	0.13
At or above 23 cm	48 (15.8)	255 (84.2)	1	1	

*Height of mother*					
Less than 150 cm	9 (42.9)	12 (57.1)	3.0 (1.24, 7.46)	**3.54 (1.07, 11.76)**	**0.039**
At or above 150 cm	79 (19.8)	320 (80.2)	1	1	

*Gestational age*					
Preterm	30 (81.1)	7 (18.9)	24 (10.07, 57.25)	**18.48 (6.51, 52.42)**	**0.000**
Term	58 (15.1)	325 (84.9)	1	1	

*Smoking during pregnancy*					
Yes	14 (46.7)	16 (53.3)	3.7 (1.75, 7.0)	**3.97 (1.59, 9.88)**	**0.003**
No	88 (21.0)	316 (81.0)	1	1	

## Data Availability

All the data of this study are available from the corresponding author upon reasonable request.
